# Effect of needle-free injection on psychological insulin resistance and insulin dosage in patients with type 2 diabetes

**DOI:** 10.3389/fendo.2024.1379830

**Published:** 2024-05-08

**Authors:** Weiping Wang, Lili Men, Yongbo Wang, Chunhong Shi, Huihui Yin, Han Li, Haicheng Zhou, Jianling Du

**Affiliations:** ^1^ Department of Endocrinology, The First Affiliated Hospital of Dalian Medical University, Dalian, China; ^2^ Dalian Key Laboratory of Prevention and Treatment of Metabolic Diseases and the Vascular Complications, The First Affiliated Hospital of Dalian Medical University, Dalian, China; ^3^ Nursing Department, The First Affiliated Hospital of Dalian Medical University, Dalian, China

**Keywords:** needle-free injection, psychological insulin resistance, type 2 diabetes mellitus, insulin aspart 30, blood glucose control, glycemic variability, insulin dosage

## Abstract

**Background and objective:**

Psychological insulin resistance (PIR), which refers to the reluctance of diabetic patients to use insulin, is a frequently encountered clinical issue. Needle-free injection (NFI) offers advantages in terms of expediting insulin absorption and mitigating adverse reactions related to injection. To evaluate the effects of subcutaneous injection of insulin aspart 30 with NFI on PIR and insulin dosage in patients with type 2 diabetes mellitus (T2DM).

**Methods:**

Sixty-four patients with T2DM participated in this randomized, prospective, open, crossover study. Insulin aspart 30 was administered subcutaneously to each subject via QS-P NFI and Novo Pen 5 (NP) successively. The effects of NFI on PIR were analyzed. Differences in insulin dosage, glycemic variability, and injection safety were compared at similar levels of glycemic control.

**Results:**

After the administration of NFI, the insulin treatment attitude scale score decreased (53.7 ± 7.3 vs. 58.9 ± 10.7, p<0.001), the insulin treatment adherence questionnaire score increased (46.3 ± 4.9 vs. 43.8 ± 7.1, p<0.001), and the insulin treatment satisfaction questionnaire score increased (66.6 ± 10.5 vs. 62.4 ± 16.5, p<0.001). At the same blood glucose level, NFI required a smaller dosage of insulin aspart 30 compared with that of NP (30.42 ± 8.70 vs. 33.66 ± 9.13 U/d, p<0.001). There were no differences in glycemic variability indices (standard deviation, mean amplitude of glycemic excursion or coefficient of variation) between the two injection methods. Compared with NP, NFI did not increase the incidence of hypoglycemia (17.2% vs. 14.1%, p=0.774), and it decreased the incidence of induration (4.7% vs. 23.4%, p=0.002) and leakage (6.3% vs. 20.3%, p=0.022) while decreasing the pain visual analog scale score (2.30 ± 1.58 vs. 3.11 ± 1.40, p<0.001).

**Conclusion:**

NFI can improve PIR in patients with T2DM and be used with a smaller dose of insulin aspart 30 while maintaining the same hypoglycemic effect.

**Clinical trial registration:**

https://www.chictr.org.cn/, identifier ChiCTR2400083658.

## 1 Introduction

With the development of the economy and society, the prevalence of diabetes is increasing. The 10th edition of the Global Diabetes Map showed that 537 million people have diabetes worldwide, and the number of adults with diabetes in China ranks first in the world (approximately 140 million), with a prevalence of 12.8% (1). At present, the treatment for diabetes is not optimal. A cross-sectional study in China revealed that 63.5% of diabetic patients received antihyperglycemic treatment, and only 35.1% of diabetic patients achieved glycemic target (2). In recent years, drugs such as glucagon-like peptide-1 receptor agonists (GLP1-RAs) and sodium-dependent glucose transporters-2 inhibitors have been introduced in China and achieved remarkable outcomes. The hypoglycemic efficacy of these medications and their capacity to mitigate the risk of diabetes complications are outstanding. However, due to reasons such as adverse reaction, high price, etc., they still cannot be universally popularized. Long-term failure to effectively control blood glucose in patients with diabetes may lead to serious complications in the heart, brain, kidney and other important organs, seriously worsening quality of life and life expectancy.

Insulin is one of the important means for controlling blood glucose in patients with diabetes. Patients with type 1 diabetes, gestational diabetes, or, in some cases, type 2 diabetes mellitus (T2DM) require insulin to control blood glucose (3). However, insulin could lead to side effects such as weight gain and hypoglycemia. In addition, subcutaneous injection may cause a fear of needles (needle phobia) and even skin damage at the injection site, such as bleeding, ecchymosis, and induration. Some diabetic patients, even medical staff, experience a psychological burden with the use of insulin and may delay or even refuse to use insulin therapy. This psychological state is called psychological insulin resistance (PIR) (4). PIR may delay the optimal time for T2DM patients to start insulin therapy. Patients who are receiving insulin therapy also have difficulty maintaining long-term adherence and satisfaction, and some patients even reduce or stop using insulin on their own. Like insulin, GLP1-RAs also face the challenge of patient reluctance towards subcutaneous injections. PIR is an important cause of the low glycemic control rate in diabetic patients and has a serious negative impact on diabetes management.

Improving diabetes education and innovations in insulin formulation and delivery methods are effective methods for improving PIR. The improvements in insulin dosage include premixed insulin and IDegAsp, which can reduce the injection frequency. Insulin injection devices have also improved, and needle-free injection (NFI) of insulin has been widely used in recent years. The principle of NFI is to shoot drugs through a small hole by instantaneous pressure generated by a mechanical device, create a high-speed jet and send drugs into subcutaneous tissue. NFI causes little damage to the skin. The pore size left by NFI on the skin surface is only 1/4 of that left by needle injection (5). NFI has limited penetration into the skin and less stimulation of nerve endings, so it can reduce pain during injection (6). Based on the above findings, needle-free injections are expected to improve PIR in diabetic patients and maintain good treatment compliance and satisfaction.

The insulin injected by NFI is dispersed in subcutaneous tissue, and its absorption efficiency is improved (7). One study revealed that NFI can accelerate the early absorption rate of rapid-acting insulin (8). Another study revealed that different skin thicknesses did not affect the absorption efficiency of needleless insulin injection (9). The efficiency of insulin absorption affects its hypoglycemic effect. Researches have shown that NFI can enhance the control of postprandial blood glucose via regular insulin and insulin aspart (10) and reduce the dose of insulin glargine needed to achieve the same blood glucose control (11).

At present, data about using premixed insulin administered by NFI in T2DM patients is scarce. Therefore, this study evaluated the effects of NFI on PIR in patients with T2DM through questionnaires and assessed whether NFI could reduce the insulin aspart 30 need of the patient through randomized, cross-controlled prospective research. The aim of this study was to provide new strategies for improving PIR and optimizing blood glucose management in patients with T2DM.

## 2 Subjects and methods

### 2.1 Patients and inclusion/exclusion criteria

This study recruited patients with T2DM from January 2021 to June 2023 at the Endocrinology Department of the First Affiliated Hospital of Dalian Medical University.

The inclusion criteria were as follows: clinical diagnosis of T2DM according to the 1999 WHO criteria; course of T2DM more than half a year; age 18-70 years; body mass index (BMI) 18-30 kg/m^2^; fasting plasma glucose (FPG) 5.0-9.0 mmol/L; glycosylated hemoglobin (HbA1c) 6.0-11.0%; use of insulin aspart 30 for at least 1 month; women of childbearing age using contraceptive measures; and normal cognitive function, ability to understand the questionnaires, and willingness to participate in the study.

The exclusion criteria were as follows: recurring hypoglycemia, diabetic ketoacidosis and other severe diabetic adverse events; severe chronic diabetic complications such as proliferative diabetic retinopathy and diabetic foot; history of pancreatitis or pancreatectomy; treatment with insulin secretagogues in the last 3 months, such as sulfonylureas; treatment with glucocorticoids or immunosuppressants; definite infection or active bleeding of important organs in the past month; acute coronary syndrome, stroke or other serious cardiovascular or cerebrovascular events in the past 6 months; anemia, dermatosis, cancer, pregnancy, breastfeeding, alcoholism, drug addiction were reasons for exclusion; laboratory examination results of alanine transaminase (ALT) or aspartate aminotransferase (AST) > 3 times the upper limit of normal or estimated glomerular filtration rate (eGFR) < 60 ml/(min*1.73m^2^).

The study was approved by the Ethics Committee of the First Affiliated Hospital of Dalian Medical University, and all the subjects signed written informed consent forms.

### 2.2 Study design

**Figure 1:**
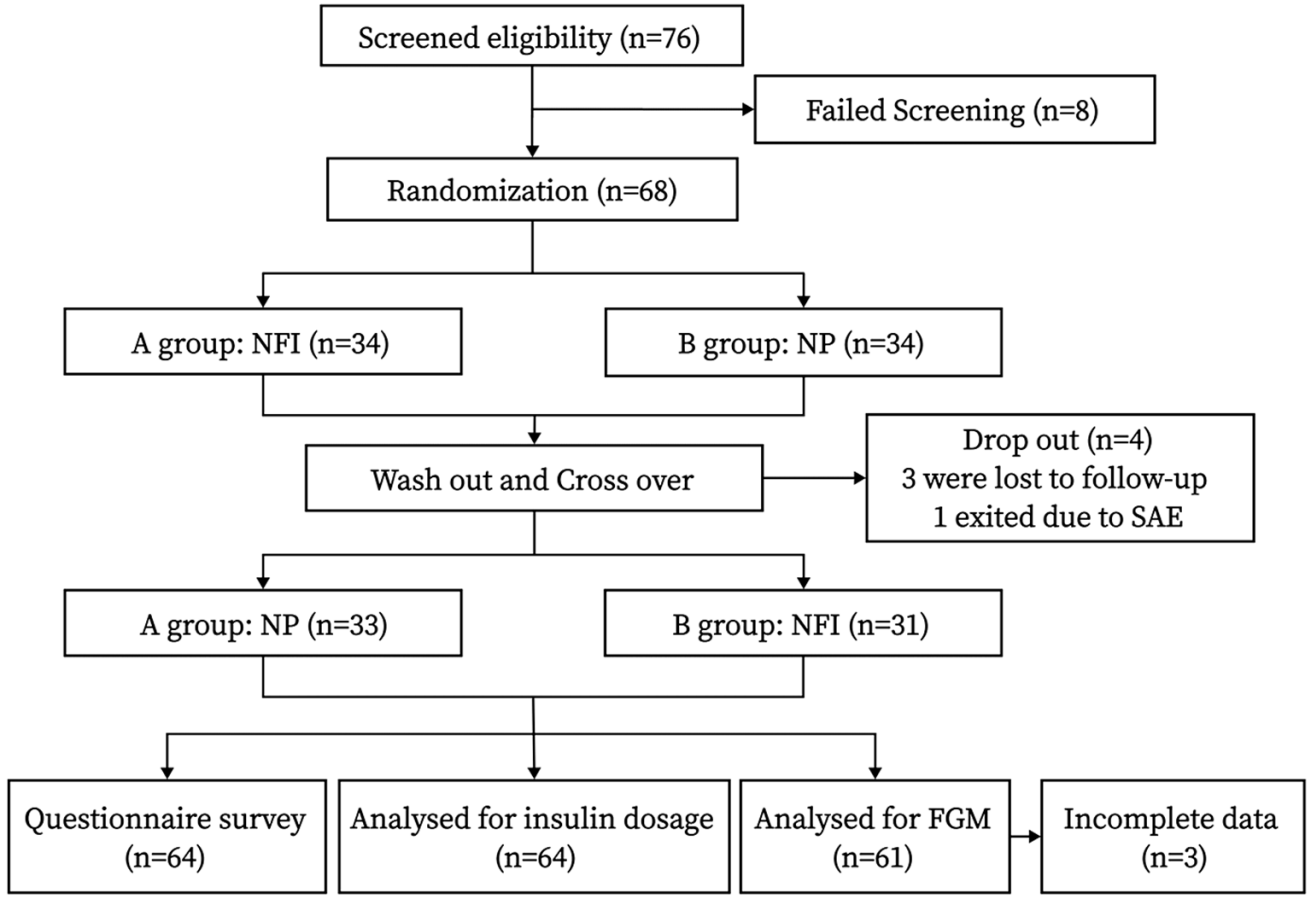
Study design. NFI, needle-free injector; NP, Novo Pen 5; FGM, flash glucose monitoring.

**Figure 2:**
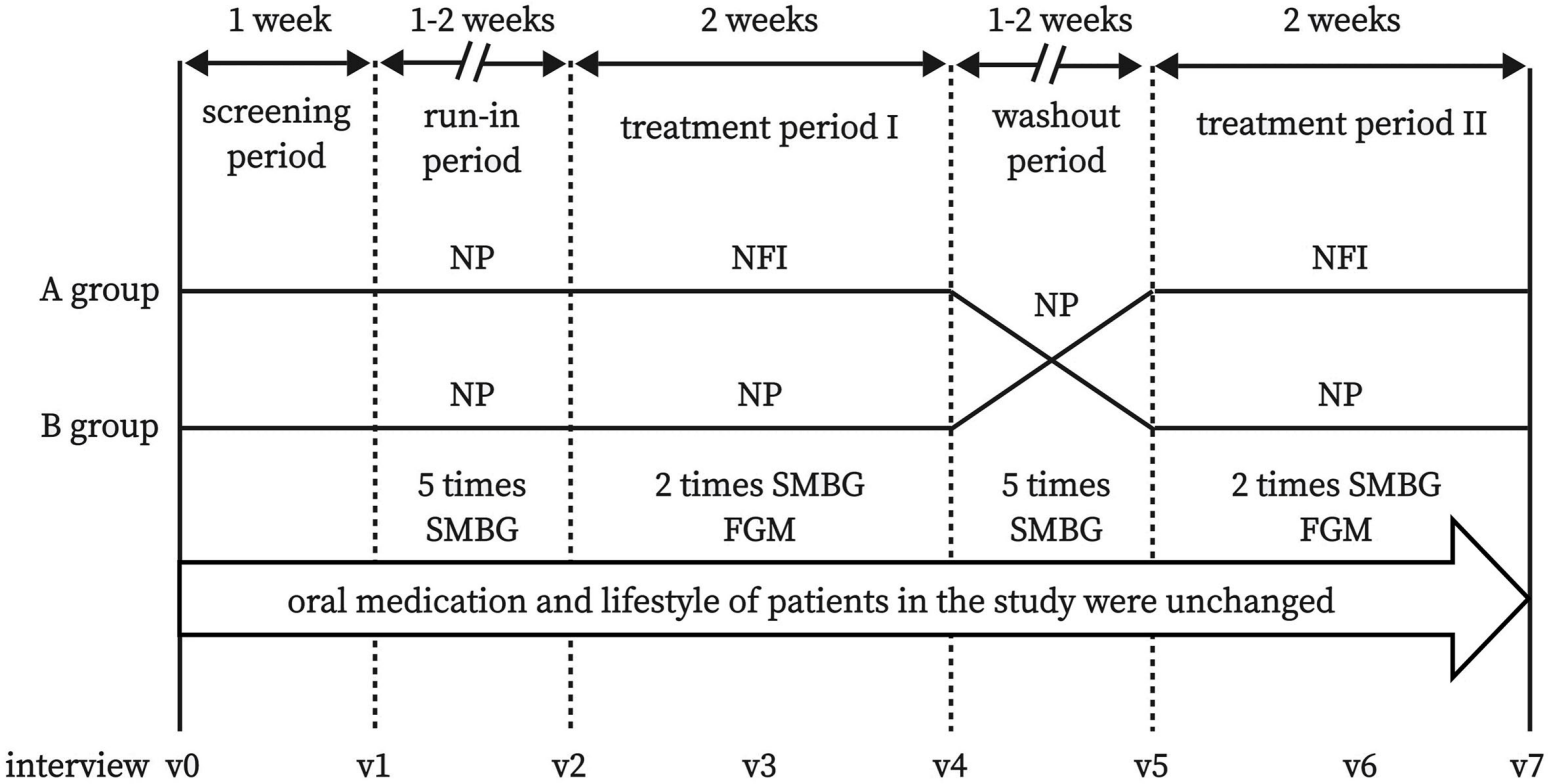
Flow chart of interview process. NFI, needle-free injector; NP, Novo Pen 5; SMBG, self-monitoring of blood glucose; FGM, flash glucose monitoring.

This was a randomized, crossover-controlled prospective study. The flow chart of the study design is shown in **Figure 1**, and the interview process is shown in **Figure 2**.

At the initial visit, the subjects were instructed on how to use NovoPen^®^ 5 (NP) (Novo Nordisk Pharmaceutical Co., Denmark) and QS-P NFI (Beijing QS Medical Technology Co., China) for subcutaneous injection of insulin aspart 30 (Novo Nordisk Pharmaceutical Co., Denmark) in the abdomen. The baseline characteristics of the subjects were collected, and questionnaires related to PIR were completed. The oral medication regimen and diet and exercise habits of the subjects remained unchanged throughout the study.

The participants were randomly assigned to group A or group B. First, the subjects entered the run-in period (1-2 weeks). NP was used in both groups, and blood glucose was monitored 5 times a day (before breakfast and dinner, as well as 2 hours after breakfast, lunch, and dinner) by self-monitoring of blood glucose (SMBG) with Accu-Chek Performa (Roche Ltd., Germany). The insulin dosage was adjusted by the researcher to ensure that the blood glucose level reached the standard (4.4-7.8 mmol/L before breakfast and dinner). After the run-in period, the subjects entered treatment period I (2 weeks). NFI was used in group A, and NP was used in group B. Blood glucose was monitored twice a day (before breakfast and dinner), and the insulin dosage adjustment is consistent with the run-in period. The FreeStyle Libre flash glucose monitoring (FGM) system (Abbott Diabetes Care, UK) was worn by a researcher to record blood glucose data during the whole treatment period I. In the washout period, both groups used NP, and blood glucose monitoring and insulin adjustment were consistent with those during the run-in period. In treatment period II, NP was used in group A, and NFI was used in group B. Blood glucose monitoring and insulin adjustment were the same as those in treatment phase I. At the end of treatment phase II, the final characteristics of the subjects were collected, and questionnaires related to PIR were completed again.

### 2.3 PIR-related investigation tools

#### 2.3.1 Insulin treatment attitude scale

This scale was used by a Chinese researcher to evaluate PIR in patients with diabetes. There are three dimensions of this scale: “misunderstanding and worry regarding insulin treatment”, “opinion about the efficacy of insulin treatment”, and “fears and limitations related to injection”. There are 20 items in the scale; each item is rated 1-5 points, and a total score >60 points indicates the presence of PIR. The Cronbach’s α coefficient of the scale was 0.88 (12, 13).

#### 2.3.2 Insulin treatment adherence questionnaire

This scale was designed by a Chinese researcher to assess insulin treatment adherence in patients with diabetes. There are 22 questions in the scale, and each question is scored 1-3 points. The questions were divided into five dimensions: “medication”, “diet”, “exercise”, “self-monitoring of blood glucose”, and “regular hospital examination”. A total score <44 indicated that the adherence to treatment was poor. The Cronbach’s α coefficient of the scale was 0.83 (13, 14).

#### 2.3.3 Insulin treatment satisfaction questionnaire

The scale, created by Anderson and translated by a Chinese researcher, is used to assess insulin treatment satisfaction in patients with diabetes. The scale consists of five dimensions: “limitations and obstacles caused by insulin injection”, “flexibility of lifestyle after insulin injection”, “confidence in avoiding symptoms caused by abnormal blood glucose”, “influence of hypoglycemia caused by insulin use on patients”, and “satisfaction with insulin injection method and its efficacy”. There are 22 questions in the scale, and each question is scored 1-7 points. The score of each dimension was converted into a percentage, and the total score of the scale was the average score of 5 dimensions. A total score <60 points indicated that the patient was dissatisfied with insulin treatment. The Cronbach’s α coefficient of the scale was 0.82 (13, 15, 16).

### 2.4 FGM system

During each treatment period, the subjects were required to wear an FGM. The FGM sensor was worn on the upper arm of the subject, and the interstitial fluid glucose concentration was recorded every 15 minutes for 14 days. The data reader was kept by the researcher, and the sensor data were not available to the subjects during the treatment period.

Blood glucose data from FGM at 3-12 days were analyzed (blood glucose data were more stable), and the data included time in range (TIR), time below range (TBR), time above range (TAR), average glucose (AG), standard deviation (SD), coefficient of variation (CV), and mean amplitude of glycemic excursion (MAGE).

### 2.5 Security and acceptance evaluation

At the end of each treatment period, the patient reported the pain on the injection site measured on a visual analog scale (VAS) ranging from 0 to 10, where 0 means no, 10 means very severe pain. Hypoglycemia (hypoglycemic symptoms or fingertip blood glucose <3.9 mmol/L) and local adverse reactions (bleeding, ecchymosis, redness, induration, and fluid leakage) were recorded during the treatment period. At the end of the study, the participants were required to complete a self- edited questionnaire to evaluate their acceptance and tolerance of NFI.

### 2.6 Collection of general characteristics and laboratory examination

At the first and last visits, general characteristics, including sex, age, duration of diabetes, medication history, smoking history, alcohol consumption history, and previous medical history, were collected. Height and weight were measured by an Omron HNH-318 (Omron Healthcare, Japan). Blood pressure was measured by an Omron HPP-1100U. Waist circumference was measured with a tape at the midpoint of the line between the lower margin of the costal arch and the iliac crest. Fasting antecubital venous blood was taken, and the indices included fasting plasma glucose (FPG), C-peptide (CP), insulin (Ins), HbA1c, ALT, AST, total cholesterol (TC), triglyceride (TG), high-density lipoprotein cholesterol (HDL-C), low-density lipoprotein cholesterol (LDL-C), uric acid (UA), and creatinine (Cre). Morning urine samples were collected to determine urinary albumin–creatinine ratio (UACR). The physical examination included an electrocardiogram.

FPG, ALT, AST, TC, TG, HDL-C, LDL-C, UA, Cre and UACR were determined by a Hitachi 7600 automatic biochemical analyzer (Hitachi, Ltd., Japan). HbA1c was determined by a Premier Hb 9210 automatic HBA1C analyzer (Trinity Biotech, Ltd., USA). A Soling LIAISON^®^XL 2210 automatic immunoanalyzer (Diasorin, Ltd., Italy) was used to determine CP and Ins via the chemiluminescence method.

### 2.7 Statistical analysis

The data analysis was performed using SPSS 22.0 software. Normally distributed data were presented as mean ± standard deviation, while non-normally distributed data were reported as median (quartiles 1 and 3). Count data were expressed as frequencies (proportions). Paired t-test was employed to compare normally distributed measurement data between groups, whereas non-parametric tests were utilized for comparing non-normally distributed measurement data between groups. Paired chi-square test was used to compare count data. Statistical significance of differences was indicated by a significance level of p<0.05.

## 3 Results

### 3.1 Patient information

A total of 76 participants were screened for the study, 68 of whom were successfully screened and randomly assigned to Group A or Group B. Ultimately, 64 participants completed the study; 33 males and 31 females were included, and the mean age was 56.91 ± 9.71 years. The general characteristics at baseline and at the end of the study are shown in **Table 1**.

**Table 1:** Characteristics of the subjects (n=64).

Characteristic	Baseline	Endpoint	*p*
Male	33 (52%)	–	–
Female	31 (48%)	–	–
Age, years	56.91 ± 9.71	–	–
Hypertension	33 (51.6%)	–	–
Coronary heart disease	15 (23.4%)	–	–
Cerebrovascular disease	11 (17.2%)	–	–
Height, cm	167.64 ± 7.56	167.72 ± 7.63	0.620
Weight, kg	72.49 ± 11.75	71.80 ± 11.7	0.740
BMI, kg/m^2^	25.72 ± 2.98	25.48 ± 2.96	0.652
WC, cm	91.87 ± 8.18	90.73 ± 8.67	0.445
SBP, mmHg	136.89 ± 18.19	132.88 ± 19.96	0.236
DBP, mmHg	76.78 ± 12.25	76.83 ± 11.60	0.982
HR, BPM	80.28 ± 10.87	79.11 ± 10.96	0.545
FPG, mmol/L	7.38 ± 1.30	7.43 ± 1.50	0.713
CP, ng/ml	1.50 (1.16,1.77)	1.89 (1.35,2.51)	0.011
Ins, mIU/L	7.44 (4.68,13.21)	9.81 (5.19,14.62)	0.106
HbA1c, %	7.81 ± 1.21	6.74 ± 0.58	<0.001
ALT, U/L	17.00 (13.00,24.50)	17.00 (13.00,22.00)	0.448
AST, U/L	19.00 (15.00,21.00)	17.00 (14.00,20.00)	0.210
TC, mmol/L	4.68 ± 1.22	4.49 ± 1.22	0.399
TG, mmol/L	1.57 (1.10,2.10)	1.47(1.15,1.95)	0.659
HDL-C, mmol/L	1.16 ± 0.28	1.14 ± 0.29	0.671
LDL-C, mmol/L	2.59 ± 0.82	2.38 ± 0.75	0.125
Cre, μmol/L	59.67 ± 15.65	62.21 ± 17.55	0.337
UA, μmol/L	336.29 ± 64.13	343.22 ± 72.95	0.493
UACR, mg/g	22.65 (12.36,55.13)	28.99 (13.91,80.13)	0.573

BMI, body mass index; WC, waist circumference; SBP, systolic blood pressure; DBP, diastolic blood pressure; HR, heart rate; FPG, fasting plasma glucose; CP, C-peptide; Ins, insulin; HbA1c, glycosylated hemoglobin; ALT, alanine transaminase; AST, aspartate aminotransferase; TC, total cholesterol; TG, triglyceride; HDL-C, high-density lipoprotein cholesterol; LDL-C, low-density lipoprotein cholesterol; Cre, creatinine; UA, uric acid; UACR, urine albumin creatine ratio. The data are presented as the mean ± standard deviation (SD), median (quartiles 1 and 3) or n (%).

### 3.2 Influence of needle-free injection on PIR

**Figure 3:**
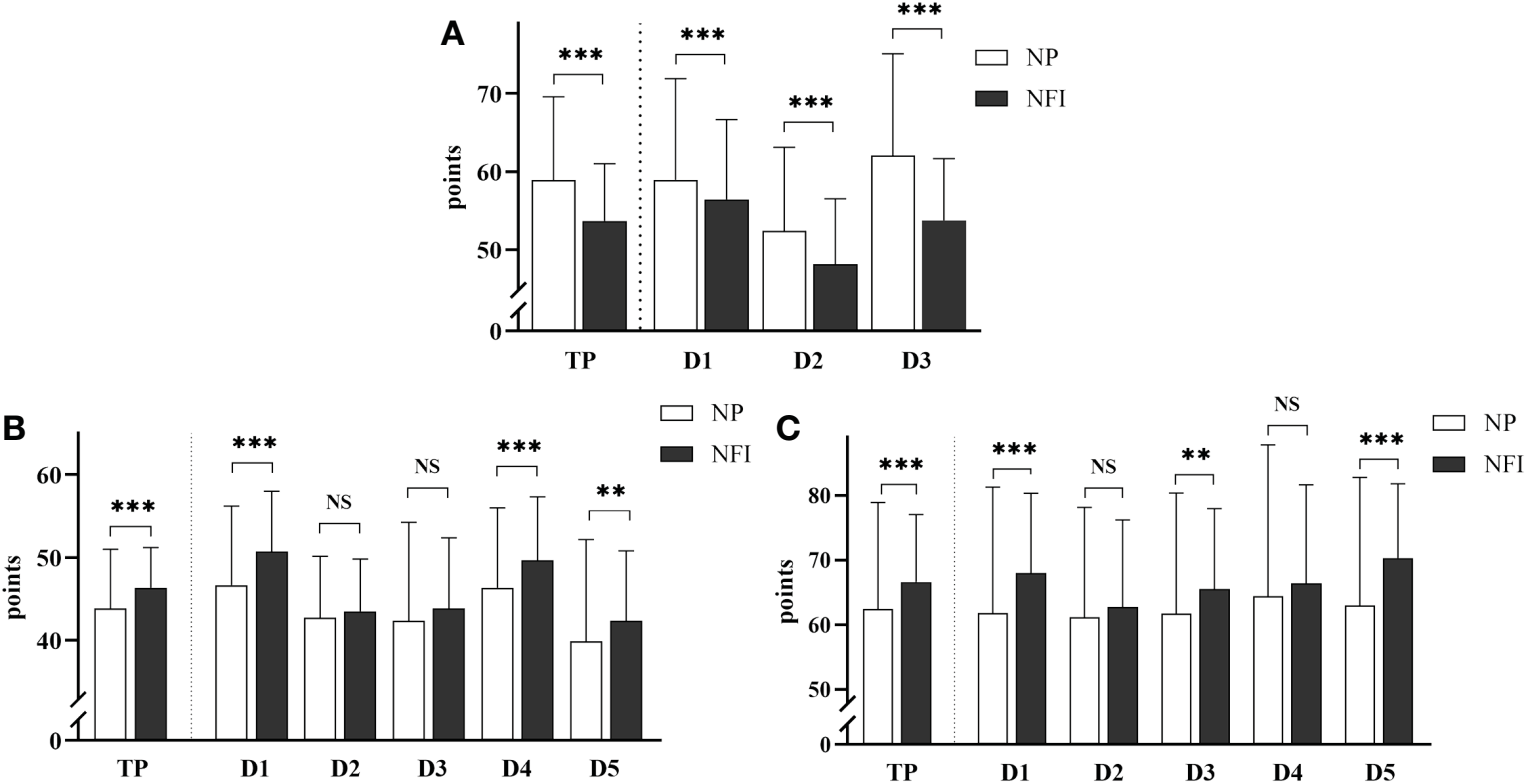
**(A)** Insulin Treatment Attitude Scale. TP: total score; D1: Dimension 1, “misunderstanding and worry about insulin”; D2: Dimension 2, “opinion about the efficacy of insulin”; D3: Dimension 3, “fears and limitations related to injection”. **(B)** Insulin Treatment Adherence Questionnaire. TP: total score; D1: Dimension 1, “medication”; D2: Dimension 2, “diet”; D3: Dimension 3, “exercise”; D4: Dimension 4, “self-monitoring of blood glucose”; D5: Dimension 5, “regular hospital examination”. **(C)** Insulin Treatment Satisfaction Questionnaire. TP is the total score; D1: Dimension 1, “limitations and obstacles caused by insulin injection”; D2: Dimension 2, “flexibility of lifestyle after insulin injection”; D3: Dimension 3, “confidence in avoiding symptoms caused by abnormal blood glucose”; D4: Dimension 4, “influence of hypoglycemia caused by insulin on patients”; D5: Dimension 5, “satisfaction with insulin injection method and its efficacy”. The data are presented as the mean ± standard deviation, and the scores for each dimension were converted to a percentage scale. NS: not significantly different; **p <0.01; ***p <0.001.

The scores of the three scales related to PIR of the two injection methods are shown in **Figure 3**. After NFI was used, the total ITAS score decreased (53.7 ± 7.3 vs. 58.9 ± 10.7, p<0.001), and the incidence of PIR decreased from 45.3% to 23.4%. The score for Dimension 3, “fears and limitations related to injection”, showed the greatest reduction. After NFI was used, the total ITAQ score increased (46.3 ± 4.9 vs. 43.8 ± 7.1, p<0.001), and the proportion of patients with poor adherence decreased from 50.0% to 29.7%. The score for Dimension 1, “medication”, increased the most. After NFI supplementation, the total ITSQ score increased (66.6 ± 10.5 vs. 62.4 ± 16.5, p<0.001), and the satisfaction with insulin increased. The score on Dimension 5, “satisfaction with insulin injection method and its efficacy”, improved most significantly.

### 3.3 Effect of NFI on insulin dosage

**Figure 4:**
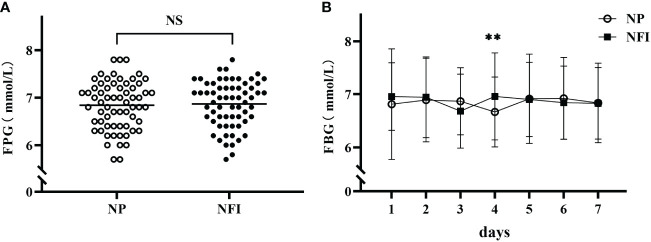
**(A)** Mean FBG levels in the last 7 days of the treatment period in the two injection methods. **(B)** Daily mean FBG during the last 7 days of the treatment period according to the two injection methods. NFI, needle-free injection; NP, Novo Pen 5. The data are presented as the mean ± standard deviation. NS, not significantly different; **p=0.003.

The fasting fingertip blood glucose (FBG) data from the last 7 days of the treatment period were relatively stable and were selected for analysis. There was no significant difference in the mean FBG between the two injection methods (6.87 ± 0.49 vs. 6.84 ± 0.51 mmol/L, p=0.265) (**Figure 4A**). There was also no significant difference in the daily mean FBG at 7 days, except on day 4 (**Figure 4B**). The above results indicated that the blood glucose control levels were the same between the two injection methods.

**Figure 5:**
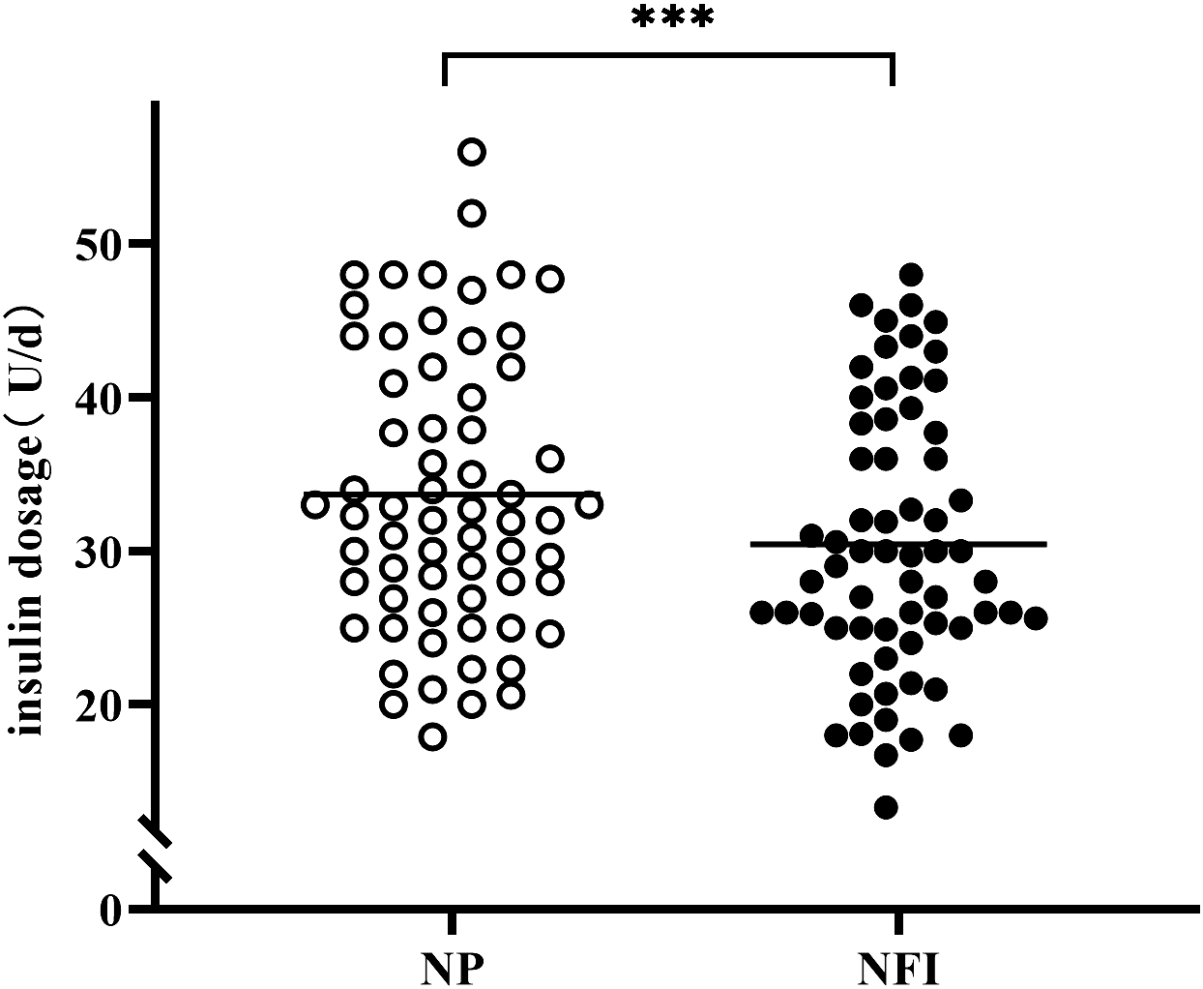
Insulin dosage of different injection methods in the last 7 days of the treatment period. NFI, needle-free injector; NP, Novo Pen 5. The data are presented as the mean ± standard deviation. ***p<0.001.

**Figure 6:**
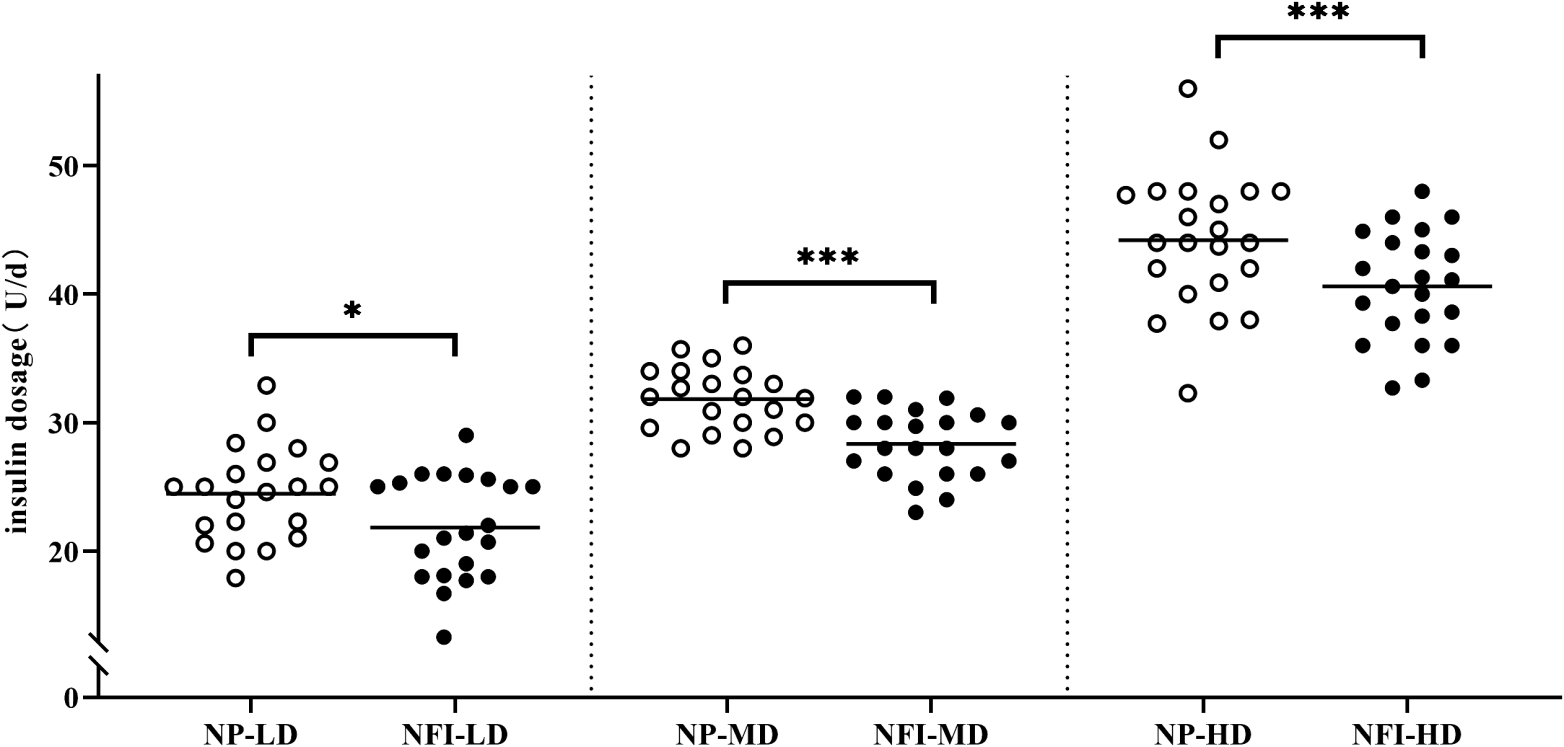
Insulin dosage in different dosage subgroups. The subjects were divided evenly into three independent subgroups (low-dose, medium-dose, high-dose) according to the daily insulin dosage. NFI, needle-free injector; NP, Novo Pen 5; LD, low-dose group; MD, medium-dose group; HD, high-dose group. The data are presented as the mean ± standard deviation. *p=0.012, ***p<0.001.

At the same blood glucose levels, the daily insulin dosage of NFI was 3.24 U lower than that of NP (30.42 ± 8.70 vs. 33.66 ± 9.13 U/d, p<0.001) (**Figure 5**). The subjects were divided into three independent subgroups (low-dose, medium-dose, and high-dose) based on tertiles of their daily insulin dosage, ranging from lowest to highest. The difference in insulin dosage between NFI and NP increases with increasing daily insulin dosage, with an average reduction of approximately 4.0 U/d in the high-dose group (**Figure 6**).

### 3.4 Glycemic variability in the FGM

Complete FGM data were obtained from 61 subjects at the end of the study (3 subjects had sensor data errors or premature shedding). Analysis of the stable FGM data from the middle 10 days of treatment period revealed that there were no significant differences in the blood glucose level indices (TIR, TAR, TBR and AG) or blood glycemic variability indices (SD, MAGE and CV) between the two injection methods (**Table 2**).

**Table 2:** FGM data for the middle 10 days of treatment under different injection methods.

Indices	NFI	NP	p
TAR, %	10.14 (3.35, 14.74)	8.23 (2.87, 15.50)	0.586
TIR, %	85.26 (82.39, 91.77)	86.79 (81.91, 91.77)	0.783
TBR, %	1.82 (0.77, 4.50)	2.01 (0.21, 5.26)	0.877
AG, mmol/L	7.12 ± 1.00	6.95 ± 1.10	0.244
SD, mmol/L	1.90 (1.70, 2.30)	1.90 (1.60, 2.20)	0.803
MAGE, mmol/L	3.85 (3.33, 4.47)	3.72 (3.29, 4.49)	0.960
CV, %	28.04 (24.56, 30.70)	28.78 (25.25, 31.56)	0.405

TAR, time above range; TIR, time in range; TBR, time below range; AG, average glucose; SD, standard deviation; MAGE, mean amplitude of glycemic excursion; CV, coefficient of variation. NFI, needle-free injector; NP, Novo Pen 5. The data are presented as the mean ± standard deviation and median (quartiles 1 and 3).

### 3.5 Safety and acceptability of needle-free injection

**Figure 7:**
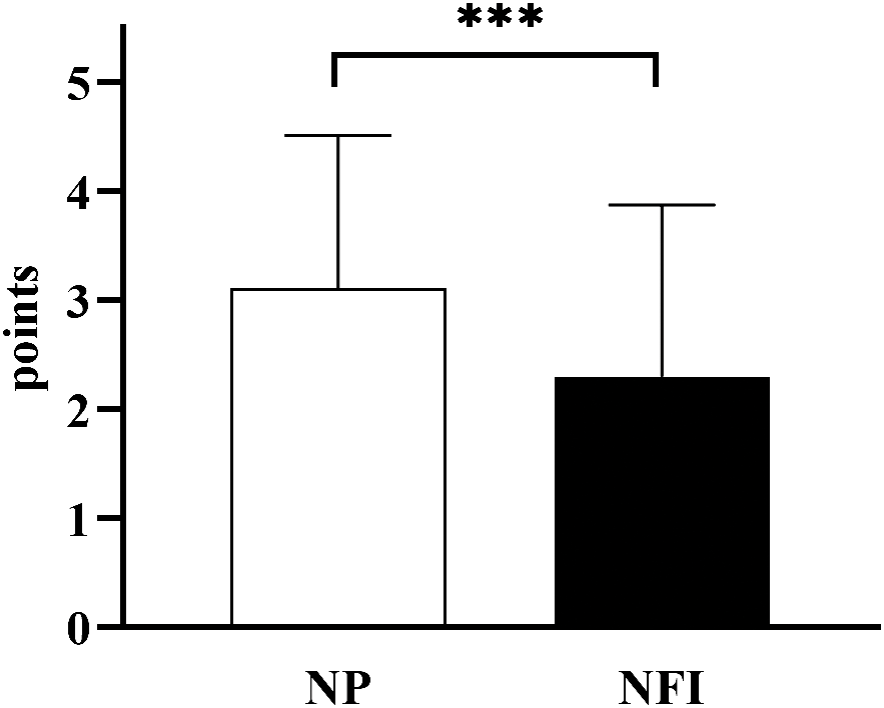
Pain VAS scores of the two injection methods. NFI, needle-free injector, NP, Novo Pen 5. The data are presented as the mean ± standard deviation. ***p<0.001.

The incidence of hypoglycemia was measured according to SMBG records (**Table 3**). A total of 17.2% of the subjects experienced hypoglycemia while using NFI, and 14.1% experienced hypoglycemia while using NP. There was no significant difference in the incidence of hypoglycemia between the two injection methods. Hypoglycemia was classified according to the Chinese Diabetes Society (CDS) guidelines: Grade 1 hypoglycemia was 3.0 mmol/L≤blood glucose <3.9 mmol/L, Grade 2 hypoglycemia was blood glucose <3.0 mmol/L, and Grade 3 hypoglycemia was hypoglycemia with serious events of consciousness and/or physical changes that required help from others without specific blood glucose limits. There was no significant difference in the frequency of hypoglycemia of different degrees between the two injection methods. In terms of injection pain, the pain VAS score of NFI was lower than that of NP (2.30 ± 1.58 vs. 3.11 ± 1.40, p<0.001) (**Figure 7**). There was no significant difference in the incidence of bleeding, ecchymosis or redness between the two injection methods. The incidence of induration and leakage in NFI-treated patients was significantly lower than that in NP-treated patients (**Table 4**). Other adverse events were assessed by baseline and endpoint characteristics (**Table 1**). There were no significant differences in vital signs (weight, BMI, WC, BP or HR) or biochemical criteria (ALT, AST, Cre, UA and UACR). No new cardiac abnormalities were found on the electrocardiogram.

**Table 3:** Incidence and severity frequency of hypoglycemia.

	Subjects with hypoglycemia	Frequency of Grade 1 hypoglycemia	Frequency of Grade 2 hypoglycemia	Frequency of Grade 3 hypoglycemia
NFI	11 (17.2%)	13	1	1
NP	9 (14.1%)	11	0	0
p	0.774	0.676	0.317	0.317

NFI, needle-free injector; NP, Novo Pen 5. The data are n or n (%).

**Table 4:** Local adverse reactions.

	bleeding	ecchymosis	redness	induration	leakage
NFI	7 (10.9%)	9 (14.1%)	8 (12.5%)	3 (4.7%)	4 (6.3%)
NP	10 (15.6%)	12 (18.8%)	8 (12.5%)	15 (23.4%)	13 (20.3%)
p	0.581	0.607	1.000	0.002	0.022

NFI, needle-free injector; NP, Novo Pen 5. The data are n (%).

The results of the self-edited questionnaire showed that 94% of the subjects were satisfied with the NFI. Most of the participants believed that NFI increased the convenience (84%) and regularity (66%) of treatment, increased confidence in controlling blood glucose (89%), and reduced fear of injection (69%). Most of the subjects believed that the occurrence of swelling (61%), induration (69%), bleeding (69%) and ecchymosis (64%) was lower after using NFI than after using NP, and 75% thought that NFI was less painful. Only 34% of the subjects believed that NFI was more affordable than NP was. Eighty percent of the participants intended to continue using NFI in the future.

## 4 Discussion

PIR is an important hindrance factor affecting the insulin utilization rate and glycemic target rate in patients with T2DM. The factors contributing to PIR originate from both patients and medical staff. Patient-related causes of PIR encompass insufficient knowledge about insulin, lifestyle inconveniences, fear of adverse reactions such as hypoglycemia, anxiety and fear associated with injections, inadequate self-efficacy in diabetes management, and limited social support (17). Medical staff-related causes involve limited experience in utilizing insulin (especially among non-endocrinologists), apprehension regarding patient rejection, concerns about the risks of hypoglycemia, reluctance to administer insulin to elderly patients,and the need for more time dedicated towards instructing injection techniques and enhancing knowledge related to insulin usage (18).

In this study, the ITAS created by a Chinese researcher was used to assess PIR of the subjects. The results showed that the incidence of PIR decreased from 45.3% to 23.4% after the use of NFI. NFI can significantly reduce the degree of PIR. The incidence of PIR investigated by the original author of the scale was 41.5%. Other studies have shown that the incidence of PIR in Chinese T2DM patients was 44.9% (19), and the incidence of PIR in other countries was 28.2-82.6% (20–22). Although the results of different studies differ greatly, the overall PIR situation is not optimistic, and NFI is expected to become an important means to improve PIR. Dimension 3, “fears and limitations related to insulin injection”, had the highest score, among which the proportion of subjects who agreed with question 18 “I am afraid that insulin injection will hurt very much” and question 19 “Even if insulin injection does not hurt much, I am still afraid to give myself injections” was the highest. This finding shows that the fear of injection itself is the main reason for patients to resist insulin. This fear may stem from pain during injection and from injection-related adverse reactions. In addition, some patients with needle phobia, while tolerating the pain of injection, are still afraid to inject themselves. The score in Dimension 3 decreased the most after NFI was used, and the results of the self-edited questionnaire also showed that most subjects believed that NFI could improve their fear of injection, which was one of the main reasons why NFI could improve PIR.

This study revealed that 50% of the participants had poor insulin compliance, which improved somewhat after the study. The adherence to Dimension 1, “medication”; Dimension 4, “self-monitoring”; and Dimension 5, “periodic review”, improved, probably due to the supervision and reinforcement of subjects in regular medication, blood glucose monitoring, and periodic review during the study period. However, the lifestyles of the subjects were solidified during the study, so there was little change in Dimension 2, “diet”, or Dimension 3, “exercise”.

In this study, the overall satisfaction of subjects receiving insulin therapy improved after NFI was administered. The satisfaction scores in Dimension 5, “satisfaction with insulin injection method and its efficacy”, Dimension 1, “limitations and obstacles caused by insulin injection”, and Dimension 3, “confidence in avoiding symptoms caused by abnormal blood glucose”, increased significantly, indicating that the subjects were more satisfied with the NFI method itself and its hypoglycemic effect. There was little change in satisfaction with Dimension 2, “flexibility of lifestyle after insulin injection”, or Dimension 4, “influence of hypoglycemia caused by insulin on patients”. It is possible that insulin treatment requires a more rigorous lifestyle and results in increased difficulty in study, work and travel, but NFI does not improve these situations. Moreover, due to the higher absorption efficiency of NFI, blood glucose levels drop relatively quickly, so additional attention is needed to prevent the occurrence of hypoglycemia.

The first-phase insulin secretion is delayed in most T2DM patients. During meals, patients need exogenous insulin to act more quickly to stabilize postprandial blood glucose. NFI can quickly stabilize early postprandial blood glucose levels due to its greater absorption efficiency (10). Previous studies on the pharmacokinetics and pharmacokinetics of NFI have mostly focused on rapid-acting insulin and insulin analogs. Premixed insulin analogs, which maintain both basal and postprandial insulin levels, have a relatively high usage rate in T2DM patients and are expected to benefit from NFI. A study revealed that, compared with that of needle-free insulin, the dosage of short-acting insulin was lower in hospitalized T2DM patients receiving intensive insulin therapy than in those receiving needle injection, but no difference was observed in long-acting insulin (23). Another study showed that NFI can reduce the dosage of insulin glargine by an average of 3.11 U/d (11). In this study, the blood glucose levels of the subjects were controlled at the same level, and the dose of insulin aspart 30 in the NFI was significantly less than that in the NP by an average of 3.2 U/d (10% of the original dose). In addition, as the insulin dosage increased, the reduction in NFI was more pronounced. In the high-dose subgroup, NFI reduced the insulin dosage by an average of approximately 4.0 U/d. A reduction in insulin dosage could alleviate injection-related adverse reactions; reduce the degree of weight gain, hypoglycemia, and hyperinsulinemia; and thus improve PIR. In addition, dosage reduction could also ease the economic burden of patients, save medical resources, and contribute to environmental protection. It can be seen that NFI has very broad development prospects.

Patients with diabetes have poor self-regulation of blood glucose. When the lifestyle is irregular or insulin is applied, the degree of glycemic variability increases significantly. SMBG is not the method to measure glycemic variability leading to potential worsening of the diabetes. In this study, FGM was used to collect blood glucose data. During each treatment period, FGM was worn for 14 days, and relatively stable blood glucose data were analyzed in the middle 10 days (days 3-12). The results showed that there was no significant difference in blood glucose levels (TAR, TIR, TBR, or AG) between the two injection methods, which confirmed that the blood glucose control level was the same between the two injection methods. No significant difference was found in the indices of glycemic variability (SD, MAGE, or CV) between the two injection methods. Other studies also did not find an effect of NFI on glycemic variability (11, 24). Although NFI could stabilize early postprandial blood glucose, it has little effect on glycemic variability.

Hypoglycemia is one of the main adverse reactions to insulin, and severe hypoglycemia is life-threatening. When insulin is absorbed relatively quickly by NFI, patients need to be more vigilant about hypoglycemia. A meta-analysis showed no difference in hypoglycemia rates between the two injections (25). The results of this study showed that the incidence and severity of hypoglycemia in NFI-treated patients were greater than those in NP-treated patients, but the difference was not significant. To avoid hypoglycemia, patients with diabetes are advised to appropriately reduce the original dosage when using needle-free syringes for the first time.

Traditional needle injection may cause pain, induration, bleeding, ecchymosis and other injection-related adverse reactions, which are also among the main causes of PIR. Improper use habits, such as reusing needles, could increase the incidence of these adverse reactions. NFI results in less damage to the skin, limited depth into the subcutaneous tissue and less stimulation to nerve endings, which could reduce these adverse reactions. A meta-analysis showed that the incidence of redness, swelling and induration after NFI was less than that after insulin injection, and there was no significant difference in the incidence of pain, bleeding or ecchymosis (25). In this study, compared with those after the use of NP, the incidence of induration and leakage after the use of NFI was significantly lower. The incidence of bleeding and ecchymosis decreased, but the difference was not significant. The pain VAS score can transform pain, a subjective experience that is difficult to quantify, into a measurable form. This study revealed that the pain VAS score was lower in NFI-treated patients than in NP-treated patients. The results of the self-edited questionnaire also showed that most subjects believed that NFI could reduce the occurrence of pain, redness, induration, bleeding and ecchymosis. In general, NFI is safer than needle injection and can alleviate resistance to insulin therapy in patients.

In terms of acceptance, the results of our self-edited questionnaire showed that most subjects were satisfied with NFI, believed that NFI could increase the convenience and regularity of treatment and increase their confidence in controlling blood glucose, and were willing to continue using NFI in the future. However, only a small number of subjects believe that NFI is more economical than NP. With NFI as an emerging technology, the price of injection devices is still not affordable for most patients, and challenges still exist in terms of popularization.

The highlights of this study are to introduce needle-free insulin injection into PIR of T2DM patients, explore the effect of NFI in improving PIR, and provide a new solution strategy for enhancing blood glucose management in T2DM patients. However, there are some limitations in this study. First of all, the sample size is not large enough, and further verification is needed to determine if the results can be applied to all T2DM patients. Additionally, this is a short-term study, and it remains unknown whether long-term use of NFI can maintain its current advantages without causing more injection-related adverse reactions.

## 5 Conclusions

NFI improved PIR in T2DM patients and increased insulin therapy adherence and satisfaction. NFI can reduce the dosage of premixed insulin analogs while achieving the same hypoglycemic effect as needle injection. NFI does not affect glycemic variability, does not significantly increase the risk of hypoglycemia, and reduces pain and injection-related adverse reactions. Therefore, the application and popularization of NFI may lead to new strategies for optimizing blood glucose management.

## Data availability statement

The raw data supporting the conclusions of this article will be made available by the authors, without undue reservation.

## Ethics statement

The studies involving humans were approved by Ethics Committee of First Affiliated Hospital of Dalian Medical University. The studies were conducted in accordance with the local legislation and institutional requirements. The participants provided their written informed consent to participate in this study.

## Author contributions

WW: Writing – original draft, Data curation, Formal analysis, Investigation, Visualization, Validation. LM: Data curation, Formal analysis, Resources, Writing – review & editing. YW: Resources, Writing – review & editing. CS: Resources, Writing – review & editing. HY: Investigation, Writing – review & editing. HL: Investigation, Writing – review & editing. HZ: Conceptualization, Investigation, Methodology, Project administration, Writing – review & editing. JD: Conceptualization, Funding acquisition, Methodology, Supervision, Writing – review & editing.

## References

[1] International Diabetes Federation. IDF diabetes atlas. 10th ed. Brussels, Belgium: IDF (2021).

[2] Ruan Y Yan QH Xu JY Yang QD Yao HH Li R. Epidemiology of diabetes in adults aged 35 and older from Shanghai, China. BioMed Environ Sci. (2016) 29:408–16. doi: 10.3967/bes2016.053 27470101

[3] Hu C Jia W. Diabetes in China: Epidemiology and genetic risk factors and their clinical utility in personalized medication. Diabetes. (2018) 67:3–11. doi: 10.2337/dbi17-0013 29263166

[4] Brod M Kongsø JH Lessard S Christensen TL. Psychological insulin resistance: patient beliefs and implications for diabetes management. Qual Life Res. (2009) 18:23–32. doi: 10.1007/s11136-008-9419-1 19039679

[5] Baxter J Mitragotri S. Jet-induced skin puncture and its impact on needle-free jet injections: experimental studies and a predictive model. J Control Release. (2005) 106:361–73. doi: 10.1016/j.jconrel.2005.05.023 16002174

[6] Sonoki K Yoshinari M Iwase M Tashiro K Iino K Wakisaka M. Regurgitation of blood into insulin cartridges in the pen-like injectors. Diabetes Care. (2001) 24:603–4. doi: 10.2337/diacare.24.3.603 11289490

[7] Engwerda EE Abbink EJ Tack CJ Galan BE. Improved pharmacokinetic and pharmacodynamic profile of rapid-acting insulin using needlefree jet injection technology. Diabetes Care. (2011) 34:1804–8. doi: 10.2337/dc11-0182 PMC3142054 21715522

[8] Hu J Shi H Zhao C Li X Wang Y Cheng Q. Lispro administered by the QS-M Needle-Free Jet Injector generates an earlier insulin exposure. Expert Opin Drug Deliv. (2016) 13:1203–7. doi: 10.1080/17425247.2016.1198772 27267431

[9] Pan Q Zhang L Gu A Yu D Wang X Zhou Y. The absorption of needle-free insulin aspart through jet injector in different body parts of healthy individuals. Front Endocrinol (Lausanne). (2022) 13:832726. doi: 10.3389/fendo.2022.832726 35574009 PMC9099202

[10] Guo L Xiao X Sun X Qi C. Comparison of jet injector and insulin pen in controlling plasma glucose and insulin concentrations in type 2 diabetic patients. Med (Baltimore). (2017) 96:e5482. doi: 10.1097/MD.0000000000005482 PMC5228650 28072690

[11] Xing Y Xie X Xu J Liu J He Q Yang W. Efficacy and safety of a needle-free injector in Chinese patients with type 2 diabetes mellitus treated with basal insulin: a multicentre, prospective, randomised, crossover study. Expert Opin Drug Deliv. (2019) 16:995–1002. doi: 10.1080/17425247.2019.1649251 31359813

[12] Ding X. Psychological Insulin Resistance survey to type 2 Diabetic patients in Guangdong Province from 2009 to 2010. Sun Yat-sen University, Guangzhou (China (2010).

[13] LI Y LI J Zhang J WU H XU Z. Study on the relationship among attitude, satisfaction and adherence with insulin treatment in patients with type 2 diabetes. J Nurs Administration. (2014) 14:633–4.

[14] Li X Cai H. Investigation on compliance behavior of diabetic patients in out-of-hospital treatment. Chin J Nurs. (2004) 39:500–1.

[15] Anderson RT Skovlund SE Marrero D Levine DW Meadows K Brod M. Development and validation of the insulin treatment satisfaction questionnaire. Clin Ther. (2004) 26:565–78. doi: 10.1016/S0149-2918(04)90059-8 15189754

[16] Li Y Xu C Xu Z Zhang J. Reliability and validity of the chinese version of insulin treatment satisfaction questionnaire. Nurs J Chin People's Liberation Army. (2013) 30:11–4.

[17] Yu JH Kim HY Kim SR Ko E Jin HY. Factors influencing psychological insulin resistance in type 2 diabetes patients. Int J Nurs Pract. (2019) 25:e12733. doi: 10.1111/ijn.12733 30945437

[18] Keqing D Chunye G Enfang F Jie Z Lin X. Investigation on psychological insulin resistance among general practitioners in Shanghai Pudong suburb community health service center. Occupation Health. (2018) 34:1843–6.

[19] Lee KP. Validity and reliability of the Chinese version of the Insulin Treatment Appraisal Scale among primary care patients in Hong Kong. Hong Kong Med J. (2016) 22:306–13. doi: 10.12809/hkmj154737 27256468

[20] Polonsky WH Fisher L Guzman S Villa-Caballero L Edelman SV. Psychological insulin resistance in patients with type 2 diabetes: the scope of the problem. Diabetes Care. (2005) 28:2543–5. doi: 10.2337/diacare.28.10.2543 16186296

[21] Hosomura N Malmasi S Timerman D Lei VJ Zhang H Chang L. Decline of insulin therapy and delays in insulin initiation in people with uncontrolled diabetes mellitus. Diabetes Med. (2017) 34:1599–602. doi: 10.1111/dme.13454 28905434

[22] Gulam AH Otieno FC Oyoo GO. Prevalence of Psychological Insulin Resistance among Patients with Type 2 Diabetes at Kenyatta National Hospital, Kenya. Health Sci J. (2017) 11:3. doi: 10.21767/1791-809X

[23] Wu Q Deng M Wang W Yu S Wang M Sun C. A self-controlled, cross-over study of intensive insulin treatment with needle-based injection versus needle-free injection in hospitalized patients with type 2 diabetes. Front Endocrinol (Lausanne). (2023) 14:1162176. doi: 10.3389/fendo.2023.1162176 37501783 PMC10369336

[24] Kong X Luo M Cai L Zhang P Yan R Hu Y. Needle-free jet injection of insulin glargine improves glycemic control in patients with type 2 diabetes mellitus: a study based on the flash glucose monitoring system. Expert Opin Drug Deliv. (2021) 18:635–41. doi: 10.1080/17425247.2021.1863945 33317342

[25] Shang J Dong M Ren Y. Meta-analysis of the efficacy and safety of needle-free syringes and insulin pens for insulin injection to treat diabetes mellitus. Chin Pharm Affairs. (2019) 33:10.

